# Responses of isoprene emission and photochemical efficiency to severe drought combined with prolonged hot weather in hybrid *Populus*

**DOI:** 10.1093/jxb/eraa415

**Published:** 2020-09-30

**Authors:** Zhihong Sun, Yan Shen, Ülo Niinemets

**Affiliations:** 1 School of Forestry and Bio-Technology, Zhejiang A&F University, Hangzhou, Zhejiang, China; 2 Zhejiang A&F University State Key Laboratory of Subtropical Silviculture, Hangzhou, Zhejiang, China; 3 Institute of Agricultural and Environmental Sciences, Estonian University of Life Sciences, Kreutzwaldi, Tartu, Estonia; 4 Estonian Academy of Sciences, Kohtu, Tallinn, Estonia; 5 University of Birmingham, UK

**Keywords:** Antioxidants, BVOC, MEP pathway, DMADP pool size, heat stress, photosynthesis, *Populus*, water stress

## Abstract

Isoprene emissions have been considered as a protective response of plants to heat stress, but there is limited information of how prolonged heat spells affect isoprene emission capacity, particularly under the drought conditions that often accompany hot weather. Under combined long-term stresses, presence of isoprene emission could contribute to the maintenance of the precursor pool for rapid synthesis of essential isoprenoids to repair damaged components of leaf photosynthetic apparatus. We studied changes in leaf isoprene emission rate, photosynthetic characteristics, and antioxidant enzyme activities in two hybrid *Populus* clones, Nanlin 1388 (relatively high drought tolerance) and Nanlin 895 (relatively high thermotolerance) that were subjected to long-term (30 d) soil water stress (25% versus 90% soil field capacity) combined with a natural heat spell (day-time temperatures of 35–40 °C) that affected both control and water-stressed plants. Unexpectedly, isoprene emissions from both the clones were similar and the overall effects of drought on the emission characteristics were initially minor; however, treatment effects and clonal differences increased with time. In particular, the isoprene emission rate only increased slightly in the Nanlin 895 control plants after 15 d of treatment, whereas it decreased by more than 5-fold in all treatment × clone combinations after 30 d. The reduction in isoprene emission rate was associated with a decrease in the pool size of the isoprene precursor dimethylallyl diphosphate in all cases at 30 d after the start of treatment. Net assimilation rate, stomatal conductance, the openness of PSII centers, and the effective quantum yield all decreased, and non-photochemical quenching and catalase activity increased in both control and water-stressed plants. Contrary to the hypothesis of protection of leaf photosynthetic apparatus by isoprene, the data collectively indicated that prolonged stress affected isoprene emissions more strongly than leaf photosynthetic characteristics. This primarily reflected the depletion of isoprene precursor pools under long-term severe stress.

## Teaser

Isoprene emission is more sensitive than photosynthesis to prolonged water and heat stress in hybrid poplar, and this reflects depletion of isoprene precursor pools under severe combined stress.

## Introduction

Isoprene is a biogenic volatile organic compound (BVOC) that is mainly emitted from a number of broadleaved temperate and tropical tree species (Guenther *et al.*, 1995, 2006; Arneth *et al.*, 2008). Due to its high abundance and atmospheric reactivity, isoprene plays important roles in the oxidative status of air at regional and global levels and in particle formation (Wilkinson *et al.*, 2009; Mentel *et al.*, 2013; Harper and Unger, 2018). As an evolutionary preserved characteristic in several plant genera, isoprene emission clearly provides a benefit to plant performance in different stressful growth environments (Guenther *et al.*, 1995, 2006; Arneth *et al.*, 2008). For example, there is evidence that isoprene improves plant thermotolerance (Sharkey *et al.*, 1996, 2001; Singsaas *et al.*, 1997; Velikova *et al.*, 2006; Behnke *et al.*, 2007; Sasaki *et al.*, 2007; Sun *et al.*, 2013b), stabilizes lipid membranes (Singsaas *et al.*, 1997; Siwko *et al.*, 2007; Velikova *et al.*, 2011), eliminates reactive oxygen species (Loreto *et al.*, 2001, 2004*b*; Affek and Yakir, 2002; Velikova *et al.*, 2005; Vickers *et al.*, 2009; Jardine *et al.*, 2012), and consumes excess reductive equivalents generated by photosynthesis (Lichtenthaler, 2000; Seemann *et al.*, 2006; Sharkey *et al.*, 2008; Sun *et al.*, 2012; Grote *et al.*, 2014).

Isoprene emission rate varies depending on instantaneous and long-term environmental conditions. There is evidence that isoprene emissions can rapidly acclimate to increases in ambient temperature, with responses ranging from seconds (e.g. Singsaas *et al.*, 1999; Sun *et al.*, 2013*a*) to a few days (e.g. Hanson and Sharkey 2001), supporting the role of isoprene in protecting plants from high temperatures. Hot weather often occurs together with drought, and such conditions can lead to a particularly severe heat stress due to reduced transpirational leaf cooling (Valladares and Pearcy, 1997; Niinemets, 2010). Moderate drought alone can either have no significant impact on isoprene emission or can stimulate it (Brüggemann and Schnitzler, 2002; Pegoraro *et al.*, 2004; Brilli *et al.*, 2007; Fortunati *et al.*, 2008; Tattini *et al.*, 2014; Bamberger *et al.*, 2017), again suggesting that isoprene might contribute to plant stress resistance. In contrast, prolonged drought often results in reduced isoprene emission (Pegoraro *et al.*, 2004; Fortunati *et al.*, 2008; Brilli *et al.*, 2013). However, studies examining acclimation of isoprene emission to rising temperatures have been relatively short-term, ranging from minutes to a few days (Singsaas and Sharkey, 1998; Hanson and Sharkey, 2001; Fortunati *et al.*, 2008), and there is currently limited information of the extent to which isoprene emission can acclimate to sustained hot weather alone and to hot weather combined with long-term drought. Long-term acclimation to high temperatures and drought also involves many other biochemical alterations, including changes in non-volatile antioxidants such as antioxidant enzymes, water-soluble antioxidants, and membrane-soluble antioxidants such as carotenoids and tocopherols (Ryan *et al.*, 2014; Tattini *et al.*, 2014, 2015; Brunetti *et al.*, 2015). Thus, study-to-study differences in isoprene emission responses to temperature might reflect a combined effect of differences in the severity and duration of the heat stress applied, and also depend on whether or not it co-occurs with other stresses. The differences might be indicative of changes in the mechanisms by which isoprene biosynthesis and emission can enhance stress resistance under different severities of environmental stress.

Isoprene formation in plants occurs via the chloroplastic 2-C-methyl-d-erythritol 4-phosphate (MEP) pathway. This pathway operates almost independently of the cytosolic mevalonic acid (MVA) pathway (Delwiche and Sharkey, 1993; Schwender *et al.*, 1997; Lichtenthaler, 2000; Affek and Yakir, 2003; Wolfertz *et al.*, 2004), although there is a certain degree of crosstalk between the two pathways at the level of the immediate isoprene precursors dimethylallyl diphosphate (DMADP) and its isomer isopentenyl diphosphate (IDP) (Kreuzwieser *et al.*, 2002; Schnitzler *et al.*, 2004; Loreto *et al.*, 2004*a*; Trowbridge *et al.*, 2012; Rasulov *et al.*, 2015). Isoprene biosynthesis starts with condensation of glyceraldehyde 3-phosphate (GAP) and pyruvate, resulting in formation of 1-deoxy-d-xylulose 5-phosphate (DXP), which is converted to DMADP/IDP in a series of ATP- and NADPH-dependent reactions. DMADP is converted to isoprene by isoprene synthase (IspS) (Lichtenthaler, 2000; Chen *et al.*, 2011; Banerjee and Sharkey, 2014; Dani *et al.*, 2014; Niinemets *et al.*, 2015). Because GAP, ATP, and NADPH come directly from photosynthesis, leaf photosynthetic metabolism is strongly linked to isoprene synthesis (Niinemets *et al.*, 1999, 2015; Lichtenthaler, 2000; Hanson and Sharkey, 2001; Sharkey and Yeh, 2001; Seemann *et al.*, 2006; Chen *et al.*, 2011; Banerjee and Sharkey, 2014; Dani *et al.*, 2014; Rasulov *et al.*, 2016, 2018).

The MEP pathway is also responsible for synthesis of essential isoprenoids of relatively large size, such as the pigment components of the photosynthetic apparatus (e.g. carotenoids, the phytol residue of chlorophylls), plastoquinone, phytohormones (e.g. gibberellic acid, abscisic acid), and antioxidants (e.g. zeaxanthin, tocopherols) (Lichtenthaler, 2000, 2007; Banerjee and Sharkey, 2014; Sharkey and Monson, 2017). Because the affinity of DMADP for geranyl diphosphate synthase, a key enzyme for the synthesis of larger isoprenoids, is about an order of magnitude greater than that of IspS, isoprene synthesis can be strongly curbed by enhanced requirements for larger isoprenoids, for example in developing or stressed leaves (Rosenstiel *et al.*, 2004; Lichtenthaler, 2007; Vranová *et al.*, 2013; Rasulov *et al.*, 2014; Harris *et al.*, 2016; de Souza *et al.*, 2018). As the synthesis of chloroplastic isoprene and of larger isoprenoids share the common substrates DMADP and IDP, the onset of production of non-volatile isoprenoid antioxidants under sustained abiotic stress can inhibit isoprene emission due to limited substrate availability.

In this study, we investigated the influences of long-term hot weather alone and in combination with severe drought on leaf isoprene emission, utilization and dissipation of photochemical energy, assimilation of carbon, and activities of antioxidant enzymes in two clones of hybrid *Populus* grown under natural variations of atmospheric temperature and humidity. We aimed to explore how isoprene emission together with photosynthetic carbon assimilation and energy production co-acclimate to increasing drought combined with natural hot weather. Based on our results, we provide a series of hypotheses of how isoprene emission can regulate the MEP pathway under stress: (1) isoprene emission relies on photosynthetic carbon and energy fluxes to maintain a certain size of the DMADP/IDP pool and to keep high activity of the enzymatic apparatus of the MEP pathway; (2) under non-stressed conditions or under moderate stress, isoprene emission is an important means of shunting excess reducing power away from photosynthesis; and (3) under prolonged or severe stresses, isoprene emissions largely decrease, but the MEP pathway stays active and maintains the DMADP/IDP pool at a level high enough for fast synthesis of isoprenoid components of the photosynthetic apparatus to replace those that have become damaged under stress.

## Materials and methods

### Plant material and experimental design

**Fig. 1.**
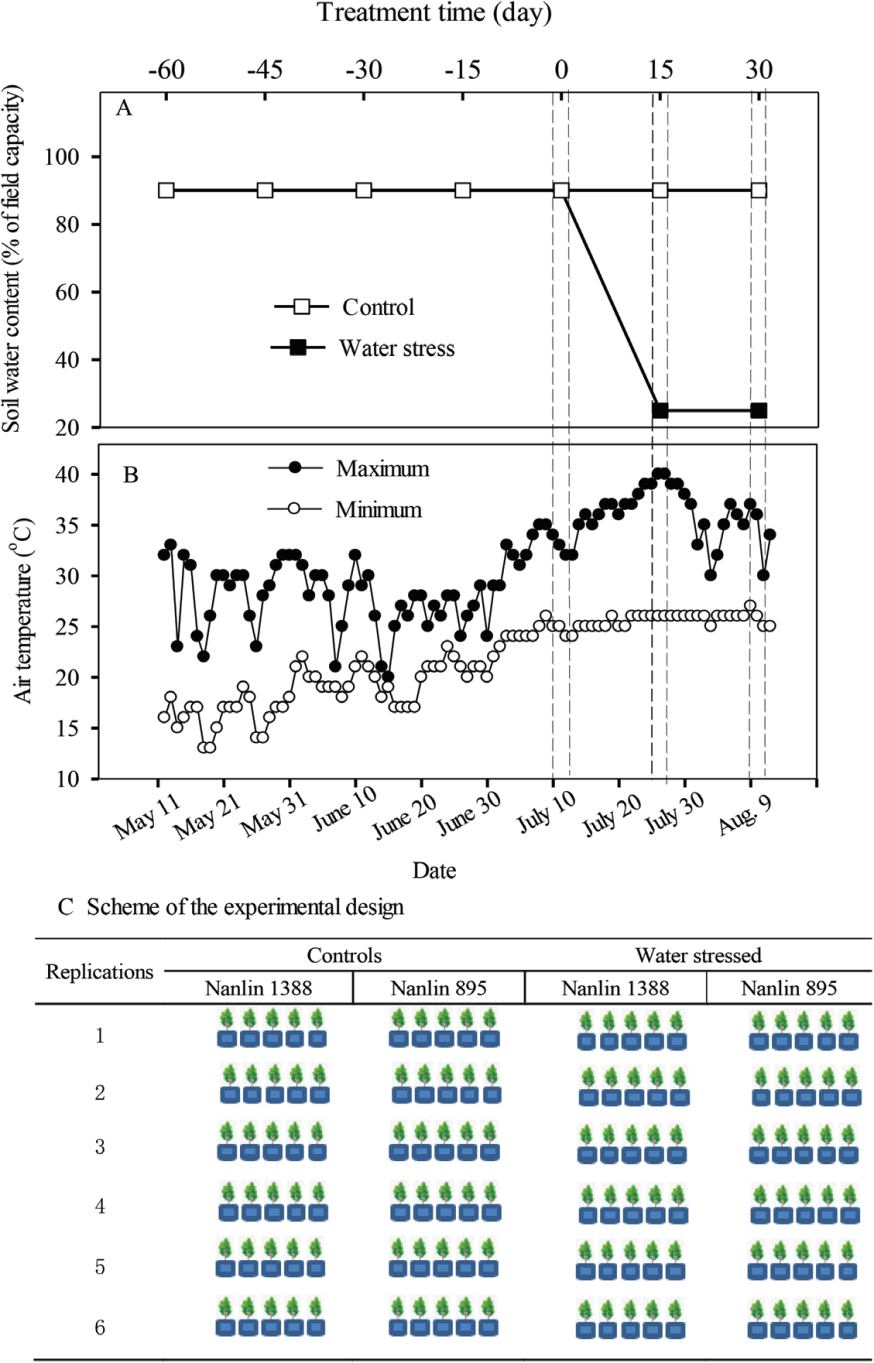
Overview of the experimental treatments and design. Details of (A) soil water content and (B) daily maximum and minimum air temperatures during the experiment. (C) Experimental design, with two clones of hybrid *Populus*, Nanlin 1388, which is tolerant to drought, and Nanlin 895, which is tolerant to heat stress. The plants were potted in 9-l pots on May 10, 2017, and soil water content was kept at >90% of field capacity till July 10, 2017 when the soil water stress treatment started. The plants in the control treatment were kept at soil water content >90% of field capacity as before, while water was withheld in the stress treatment until soil water content decreased gradually to 25% of field capacity (reached at day 15 since the start of the treatment). The soil water content was then maintained at 25% of field capacity for further 15 days till the end of the experiment. The soil drought treatment was accompanied with a natural spell of unusually hot weather (July 10 - end of the experiment). (This figure is available in colour at *JXB* online.)

One-year-old cuttings of the hybrid *Populus* clones Nanlin 1388 and Nanlin 895 were obtained from the Poplar Research and Development Center, Nanjing Forestry University. Both clones are crosses between the cultivars of *P. deltoides* Bart. × *P. euramericana* (Dode) Guineir (Wang *et al.*, 2016) and both are fast-growing and have a high isoprene emission capacity. Nanlin 1388 has high tolerance of drought and Nanlin 895 has high tolerance of heat stress (Wang *et al.*, 2016). In March 2017, cuttings of 10–15 cm long from the 1-year-old plant stems of both clones were planted in a commercial growth substrate consisting of peat, perlite, and roseite (2:1:1 by volume; JinHai Agriculture Sci & Tec Co. Ltd, Hangzhou, China) at the Zhejiang Agricultural and Forestry University (Lin’an, Hangzhou). In May, after ~40 d of growth, 20-cm tall plants were selected and transplanted into 9-l pots filled with a mixture of local field surface-soil and commercial substrate (2:1 by volume) and moved into a shed shaded by a vinyl mesh. Prior to transplanting, we measured the soil field capacity of the pots (saturated water content relative to soil dry mass, as determined after drying at 105 °C for 12 h) to be able to monitor and control the soil water content during the experiment. The cuttings were supplied with tap-water every day and the pot soil water content was maintained at 90% of the field capacity (Fig. 1A). After 45 d of growth (5 July) 60 healthy and uniform plants of both Nanlin 1388 and Nanlin 895 were selected for the experiments. During the period of seedling growth before 9 July, the weather conditions were usual for the site with air temperatures similar to previous years (Fig. 1B, Supplementary Table S1, available at *JXB* online).

The experiment was conducted using a factorial design with four clone × treatment combinations: Nanlin 1388 well-watered control (C_Nanlin1388_), Nanlin 895 well-watered control (C_Nanlin895_), Nanlin 1388 drought treatment (WS_Nanlin1388_), and Nanlin 895 drought treatment (WS_Nanlin 895_). For each treatment, six replicates were used, each of which consisted of five individual cuttings. Hence, there were 30 individual cuttings for each clone × treatment combination and a total of 120 individual cuttings were used in the experiment (Fig. 1C).

The soil water treatments started on 10 July when a period of hot weather had just begun but had not yet influenced leaf physiological and biochemical traits. The pots were weighed every afternoon throughout the experiment to monitor the soil water status. The controls were watered daily to keep them at 90% of field capacity throughout the experiment (Fig. 1A). In the stress treatment, watering was withheld until the soil water content reached 25% of soil capacity (25 July, day 15 after the start of treatment). Thereafter, water was added as required to maintain the target of 25% field capacity for a further 15 d.

During the experiment, both the control and drought treatments were subjected to a natural period of continuous extreme hot weather (Fig. 1B, Supplementary Table S1). The first series of measurements was conducted on 9–10 July when the maximum daily temperatures were moderately high (30–33 °C). The second series of measurements was carried out on 24–25 July after a continuous period of 10 d of hot weather when the daily maximum temperature reached 40 °C. The third series of measurements was carried out on 7–9 August at the end of the heatwave when the daily maximum air temperature was 30–37 °C (Fig. 1B). Night-time temperatures were ~5–7 °C higher during the measurement period than before the start of the experiment. For each series of measurements, leaf gas-exchange, isoprene emission, and chlorophyll fluorescence characteristics were determined. To avoid the influence of leaf age, at each of the three measurement times, we selected fully mature, non-senescent leaves with similar ages of 15–20 d from upper branches (5th to 8th position below the apical bud). Leaf samples for determination of antioxidant enzyme activities were taken from the same positions in the canopy. Each sample consisted of 20–30 fresh leaf discs obtained with a stainless steel cork-borer (diameter 15 cm). The leaf discs were immediately frozen in liquid nitrogen, and stored at –80 °C until analysis.

### Measurements of leaf gas-exchange characteristics and isoprene emissions

A GFS-3000 portable gas-exchange system (Walz GmbH, Effeltrich, Germany) with an 8-cm^2^ (2×4 cm) standard leaf cuvette was used to measure the following leaf gas-exchange characteristics: net assimilation rate (*A*_n_), stomatal conductance to water vapor (*g*_s_), and intercellular CO_2_ concentration (*C*_i_). A high-sensitivity proton transfer-reaction–quadrupole mass-spectrometer (HS PTR-QMS 500, Ionicon, Innsbruck, Austria) was used for online measurements of the emission rate of isoprene, detected as the ion signal with a mass/charge (*m*/*z*) ratio of 69^+^. The PTR-QMS was connected to the gas-exchange system via a Teflon T-connection tube, allowing simultaneous measurements of gas-exchange and isoprene emission characteristics as described in Niinemets and Sun (2014). Prior to the measurements, the PTR-QMS was calibrated using a standard gas containing 15 different volatiles including isoprene (Restek Corporation, Bellefonte, PA, USA). The mixing ratio of each individual volatile was 1 ppm. The instrument was operated with the following settings: *E/N* = 130 Td (where *E* is the electric field and *N* is the number density of drift tube molecules), drift tube pressure of 2.30 mbar, inlet temperature of 60 °C, [O^2+^] and [NO^+^] < 2% of [H_3_O^+^].

All measurements were carried out under the following standard conditions: air-flow rate of 750 μmol s^−1^, leaf temperature of 30 °C, relative humidity of 60%, CO_2_ concentration of 400 µmol mol^−1^, and light intensity of 1000 μmol m^−2^ s^−1^. Before leaf enclosure, the background isoprene concentration in the air was measured under the standard conditions. The measurement leaf was then enclosed in the cuvette and allowed to stabilize under the standard conditions until the stomata were fully open and photosynthesis, transpiration, and isoprene emission rates reached a steady state, which typically took 10–20 min. Once the steady-state conditions were established, the gas-exchange characteristics and isoprene emission rate were determined. The background concentration of isoprene as measured in the empty cuvette was subtracted from the value recorded for each leaf to obtain the isoprene emission rate (*I*).

### *In vivo* estimation of the pool size of dimethylallyl diphosphate and isoprene synthase rate constant

The pool size of the immediate substrate for isoprene synthesis, dimethylallyl diphosphate (*S*_DMADP_), and the isoprene synthase (IspS) rate constant (*K*) were estimated using the method of post-illumination isoprene emission (Rasulov *et al.*, 2009, 2011). This method is based on the fact that after light is switched off, isoprene emission continues in the darkness for ~150–200 s at the expense of DMADP and IDP that have been synthesized in the light (Zhou *et al.*, 2013), and the integral of the dark emission of isoprene characterizes *S*_DMADP_ whilst the rate of the dark decay of the DMADP pool characterizes *K* (Rasulov *et al.*, 2009, 2011). There is a secondary rise in isoprene emission between ~200–600 s after darkening, but this relies on *de novo* synthesis of DMADP from upstream metabolites of the MEP pathway (Li *et al.*, 2011; Rasulov *et al.*, 2011; Li and Sharkey, 2013). Hence, after the leaf gas-exchange and isoprene emission rates had reached a steady state, the light was switched off and isoprene emission was measured for 200–300 s until a minimum emission rate, close to the baseline level, was observed. The DMADP pool size was estimated as the integral of the post-illumination isoprene emission using the extrapolated baseline and considering for the leaf chamber effect, as in our previous studies using the Walz GFS-3000 system (Sun *et al.*, 2012, 2013*a*; Niinemets and Sun, 2015; Niinemets *et al.*, 2015). The IspS rate constant was estimated by pairwise linear regression of the values of the isoprene emission rate and the available DMADP pool size at different time-points after leaf darkening.

The percentage of carbon lost from photosynthesis due to isoprene emission (*C*_isoprene_) was calculated as:

$$\begin{array}{l} C_{isoprene}=\;\left(6I/A_{n}\right)\times 100 \end{array}$$ (1)

where both *I* and *A*_n_ are in the same molar units, and the isoprene (C5 compound) emission rate is multiplied by 6 because one additional molecule of CO_2_ is released when 1-D-deoxyxylulose 5-phosphate is formed from glyceraldehyde phosphate and pyruvate.

### Measurements of chlorophyll fluorescence and relative chlorophyll content

Leaf chlorophyll fluorescence was measured using a pulse-amplitude modulated fluorimeter (PAM-2500, Walz). Before the measurements, the selected leaf was dark-adapted for 30 min using a dark-leaf clip. A low red measurement (pulse-amplitude modulated) light (<1 μmol m^−2^ s^−1^) was switched on and the fluorescence characteristics were recorded using the measurement protocol of saturation pulse analysis (Genty *et al.*, 1989; Bilger and Björkman, 1990; Kramer *et al.*, 2004). First, the minimum fluorescence yield of the dark-adapted leaf (*F*_o_) was measured, then a saturated light pulse of 8000 μmol m^−2^ s^−1^ was given for 500 ms and the maximum dark-adapted fluorescence yield (*F*_m_) was measured. A moderately high actinic light (197 μmol m^−2^ s^−1^) was switched on, and saturating light pulses were given every 20 s for 5 min, and the maximum (*F*_m_´) and steady-state (*F*) fluorescence yields were obtained. From these measurements, the following variables were calculated: the fraction of open PSII centers, assuming the centers are interconnected (*q*_L_, a measure of the degree of oxidation of the primary plastoquinone electron acceptor *Q*_A_; Kramer *et al.*, 2004), non-photochemical quenching (NPQ; Bilger and Björkman, 1990; Kramer *et al.*, 2004), and the effective photochemical quantum yield of PSII (Y(II), Genty *et al.*, 1989).

$$\begin{array}{l} q_{L}=\frac{(F_{m}{}^{\prime }-F)F_{o}{}^{\prime }}{(F_{m}{}^{\prime }-F_{o}{}^{\prime })F} \end{array}$$ (2)

$$\begin{array}{l} NPQ\;=\frac{\left(F_{m}-{F_{m}}^{\prime }\right)}{{F_{m}}^{\prime }}, \end{array}$$ (3)

$$\begin{array}{l} Y\left(II\right)\;=\frac{\left({F_{m}}^{\prime }-F\right)}{{F_{m}}^{\prime }}. \end{array}$$ (4)

The relative chlorophyll content was determined with a SPAD 502 Plus handheld chlorophyll meter (Konica Minolta) from measurements of leaf transmittance at two wavelengths (650 nm and 940 nm; Uddling *et al.*, 2007). 

### Measurement of superoxide dismutase, peroxidase, and catalase activities

Frozen leaf discs of 0.3 g were homogenized in liquid N_2_ in a mortar, and enzymes were extracted on ice with potassium phosphate buffer (0.05 mol l^−1^, pH 7.8). The homogenates were transferred to 10-ml glass tubes, brought to a volume of 8 ml, and then centrifuged (centrifuge model 5430R, Eppendorf AG, Hamburg, Germany) at 6290 *g* for 15 min at 4 °C. The supernatant was collected and kept at 4 °C for the assays. Distilled water was used for the blank controls.

Total superoxide dismutase (SOD) activity was assayed using the hydroxylamine method by employing a commercial SOD Assay Kit (A001-1-1, Nanjing Jiancheng Bioengineering Institute, China) according to the manufacturer’s instructions. The method is based on inhibition of O_2_^−^-dependent reduction of nitro blue tetrazolium (NBT) by SOD; in the reaction mixture, the xanthine/xanthine oxidase system is used as a superoxide generator (Lee *et al.*, 2001). Reaction solution mixtures of 7 ml were made from the reagents of the SOD Assay Kit, and 1 ml of reaction solution mixtures were used in analyses. For SOD measurements, 0.1 ml centrifuged leaf homogenate was used. For a zero control, 0.1 ml distilled water was added. After incubation for 40 min at 37 °C, the absorbance of the reaction mixture was measured at 550 nm using a double-beam UV6300 spectrophotometer (MAPADA Shanghai, Co. LTD, China). The total SOD activity was expressed as U g^−1^ tissue fresh mass (FM), where U is defined as the amount of enzyme that inhibits half the reaction of O_2_^−^ with NBT.

The peroxidase (POD) activity assays were conducted using the method of *o*-methoxyphenol (guaiacol) oxidization, leading to formation of a colored heterodimeric product (Doerge *et al.*, 1997) with an absorption peak at 470 nm (Dhindsa *et al.*, 1981; Abassi *et al.*, 1998). The reaction solution mixture was made by adding 0.028 ml *o*-methoxyphenol to 50 ml phosphate buffer (0.1 mol l^−1^, pH 6.0) in a beaker and heating it on a magnetic stirrer until it dissolved completely. After cooling to room temperature, 0.019 ml of 30% (w/v) H_2_O_2_ was added, and the reagent mixture was brought to 100 ml with phosphate buffer. The reaction was started by adding 1 ml POD enzyme extract to 3 ml of reaction solution mixture. Absorbance relative to the reagent mixture without POD extract (reagent mixture + 1 ml phosphate buffer, pH 7.8) was measured using the UV6300 spectrophotometer at 470 nm. Absorbance was recorded every 30 s for 3 min, and the mean rate of absorbance change (∆*A*_470_, AU min^−1^) was used to calculate the enzyme activity. The POD enzyme activity unit (U) was defined as an increase of ∆*A*_470_ of 0.1 AU min^−1^, and activity was expressed as U g^−1^ FM.

Catalase (CAT) activity was assayed using the method of Dhindsa *et al.* (1981), which is based on the measurement of changes in H_2_O_2_ concentration in a solution following addition of CAT as determined by absorbance at 240 nm. Briefly, 0.1 mol l^−1^ H_2_O_2_ standard solution in phosphate buffer (0.2 mol l^−1^, pH 7.8) was prepared by adding 5.68 ml 30% H_2_O_2_ and 3.161 g KMnO_4_ to 1000 ml distilled water. The reaction was started by adding 0.2 ml CAT enzyme extract to 2 ml of the H_2_O_2_ solution, and the absorbance at 240 nm was measured every 30 s for 3 min using the UV6300 spectrophotometer. The mean absorbance change per min (∆*A*_240_, AU min^−1^) was calculated. We defined the CAT enzyme activity unit (1 U) as 0.1 AU min ^−1^ change of ∆A_240_, and CAT activity was expressed as U g^−1^ FM.

### Statistical analysis

Three-way analysis of variance (ANOVA) was employed to analyse the impact of the main factors, clone, soil water treatment, and treatment time (degree of drought), and their interactions on all leaf physiological and biochemical traits. Differences among trait means at a given measurement time were separated using one-way ANOVA followed by Duncan’s multiple range test. Linear and non-linear regressions were used to examine correlative relationships between physiological and biochemical characteristics. ANOVAs were conducted with SPSS 17.0 (IBM Corporation, New York, NY, USA), and all plots and linear and non-linear regression analyses were made with SigmaPlot 12.5 (Systat Software, London, UK). All statistical tests were considered significant at *P*<0.05.

## Results

### Responses of isoprene emission characteristics to drought under long-term hot weather

**Fig. 2.**
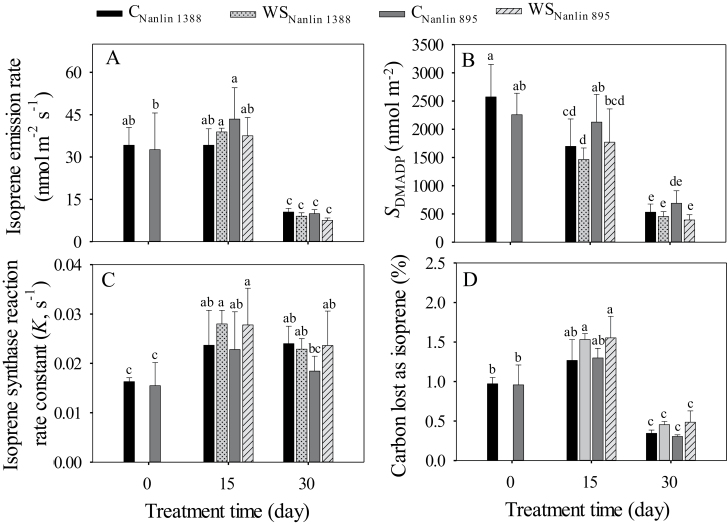
Effects of water stress on leaf isoprene emission characteristics in two hybrid poplar clones, Nanlin 1388 (tolerant to water stress) and Nanlin 895 (tolerant to heat stress). C_Nanlin 1388_ and C_Nanlin 895_ are controls, and WS_Nanlin 1388_ and WS_Nanlin 895_ are water-stressed; Fig. 1 for the experimental design and environmental conditions during the experiment). (A) Isoprene emission rate, (B) size of pool of DMADP, (C) isoprene synthase reaction rate constant, and (D) percentage of carbon lost as isoprene. During the measurements, leaf temperature was set to 30 °C, ambient CO_2_ concentration to 400 μmol mol^−1^, and light intensity to 1000 μmol m^−2^ s^−1^. Data are means (± SD), *n* = 4–6. Different letters indicate significant differences between means as determined by one-way ANOVA followed by Duncan’s multiple range test (*P*<0.05).

Although the two hybrid *Populus* clones had different abiotic stress resistance, their isoprene emission characteristics responded similarly to drought combined with long-term hot weather (Table 1, Fig. 2). Most isoprene emission characteristics were not significantly affected by water stress alone, except for a significant main effect of drought on the percentage of photosynthetic carbon lost due to isoprene emission (*C*_isoprene_; Table 1). At 15 d from the start of the drought treatment, neither the isoprene emission rate (*I*) nor the size of the DMADP pool (*S*_DMADP_) differed significantly among the control and drought-stressed plants, except for the drought-tolerant clone Nanlin 1388 (Fig. 2A, B). Isoprene emission characteristics were primarily affected by the length of the treatment, and at 30 d when drought interacted with hot weather both *I* and *S*_DMADP_ were considerably reduced in both control and drought-stressed plants (Fig. 2A, B). In contrast, the isoprene synthase (IspS) rate constant, *K*, was increased at both 15 d and 30 d after the start of the treatments (Fig. 2C), except in the controls of the thermotolerant clone Nanlin 895 at 30 d. Compared with the beginning of the treatments, *C*_isoprene_ was increased after 15 d in both the control and water-stressed plants, but it was reduced at 30 d (Fig. 2D).

**Table 1.** Main and interactive effects of water stress treatment, time of treatment and clone on leaf physiological and biochemical characteristics in two hybrid poplar clones according to three-way ANOVA

Traits		*F*_C_	*F*_W_	*F*_T_	*F*_C*W_	*F*_C*T_	*F*_W*T_	*F*_C*W*T_
Isoprene emission characteristics	*I*	0.1ns	0.0ns	94.9***	0.2ns	1.1ns	0.4ns	2.3ns
	*S*_DMADP_	0.2ns	1.2ns	139.4***	0.4ns	4.7*	2.7ns	0.1ns
	*K*	1.6ns	3.2ns	31.9***	0.6ns	0.2ns	2.0ns	1.1ns
	*C*_isoprene_	0.0ns	11.8**	105.1***	1.4ns	0.5ns	0.5ns	1.0ns
Gas-exchange characteristics	*g*_s_	1.6ns	46.2***	131.7***	0.14ns	0.29ns	11.8**	0.52ns
	*C*_i_	2.7ns	40.2***	79.1***	2.5ns	0.34ns	17.9**	0.63
	*A*_n_	0.3ns	98.4***	80.1***	9.8**	1.3 ns	16.1***	0.5ns
Chlorophyll fluorescence traits and relative chlorophyll content	*q*_L_	3.2ns	83.4***	53.8***	8.5**	2.9ns	8.9**	0.3ns
	Y(II)	1.7ns	170.2***	34.1***	5.6*	1.7ns	3.1ns	0.64ns
	NPQ	0.15ns	64.3***	35.4***	0.09ns	2.9ns	0.51ns	4.8*
	*R*_Chl_	7.6**	0.47ns	14.8***	1.1ns	7.2**	3.8ns	2.4ns
Antioxidant enzyme activities	SOD	9.6**	6.8**	20.2***	0.7ns	2.0ns	15.6***	0.07ns
	POD	82.4***	0.0ns	1.2ns	0.30ns	1.9ns	1.8ns	1.6ns
	CAT	0.12ns	17.7***	22.6***	0.20ns	0.43ns	6.6**	0.90ns

Details of experimental conditions are given in Fig. 1. Data are *F*-values according to three-way ANOVA. Subscripts as: C, clone (Nanlin 1388 and Nanlin 895); W, water stress treatment; T, time (duration) of treatment (measurements taken at 0, 15, and 30 d). The start of the water stress treatment coincided with a heat spell that lasted 12 d. Traits: *I*, isoprene emission rate; *S*_DMADP_, size of the pool of dimethylallyl diphosphate (DMADP); *K*, isoprene synthase activity; *C*_isoprene_, percentage of carbon lost due to isoprene emission; *g*_s_, stomatal conductance to water vapour; *C*_i_, intercellular CO_2_ concentration; *A*_n_, net assimilation rate; *q*_L_, photochemical quenching; Y(II), effective PSII quantum yield; NPQ, non-photochemical quenching; *R*_Chl_, relative chlorophyll content (SPAD value).

There were 4–6 independent replicates for each treatment for gas-exchange, isoprene emission characteristics and antioxidant enzyme activities, and 12–18 replicates for chlorophyll fluorescence traits and relative chlorophyll content for each treatment at each treatment time. * - *P*<0.05, ** - *P*<0.01, *** - *P*<0.001; ns, not significant.

### Changes in leaf gas-exchange and chlorophyll fluorescence traits under drought and long-term hot weather

**Fig. 3.**
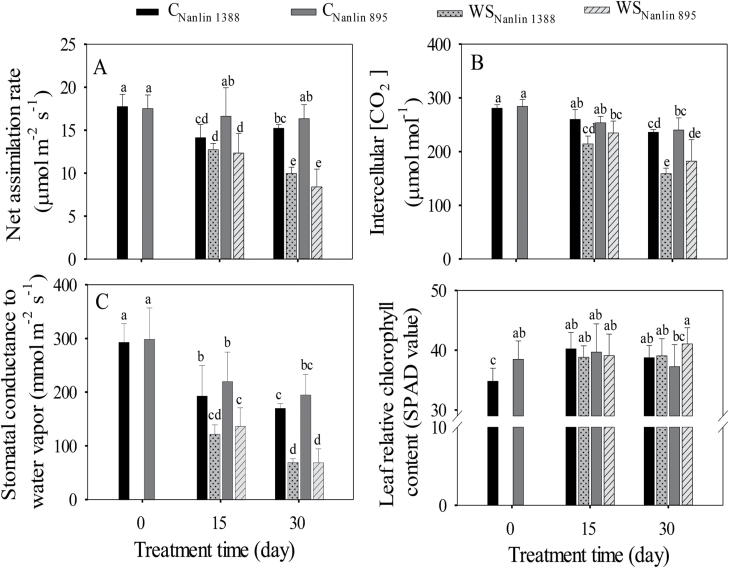
Effects of water stress on leaf gas exchange characteristics in two hybrid poplar clones, Nanlin 1388 (tolerant to water stress) and Nanlin 895 (tolerant to heat stress). C, control, WS, water stress (Fig. 1 for the experimental design and environmental conditions during the experiment). (A) Net assimilation rate, (B) intercellular CO_2_ concentration, (C) stomatal conductance, and (D) leaf relative chlorophyll content (SPAD values). During the measurements, leaf temperature was set to 30 °C, ambient CO_2_ concentration to 400 μmol mol^−1^, and light intensity to 1000 μmol m^−2^ s^−1^. Data are means (± SD), *n* = 4–6 for gas exchange and *n* = 12–18 for chlorophyll. Different letters indicate significant differences between means as determined by one-way ANOVA followed by Duncan’s multiple range test (*P*<0.05).

The responses of photosynthetic characteristics to the treatments were similar in both clones (Table 1, Fig. 3). The drought treatment and treatment time significantly affected all leaf gas-exchange characteristics, and reductions in *A*_n_, *C*_i_, and *g*_s_ typical of drought-stressed plants became more severe as drought progressed (Table 1, Fig. 3A–C). In addition, *A*_n_, *C*_i_, and *g*_s_ in the well-watered plants were also reduced after 15 d and 30 d of treatment, except for *A*_n_ in Nanlin 895 at both measurement dates (Fig. 3A) and *C*_i_ in both clones after 15 d (Fig. 3B). The interactive effect of the drought treatment × time was always significant, primarily indicating that the time-dependent reductions in photosynthetic characteristics became stronger in drought-stressed plants as drought progressed (Table 1, Fig. 3A–C). In contrast to the photosynthetic characteristics, relative leaf chlorophyll content (SPAD value) was little affected throughout the experiment, except for an increase observed in Nanlin 1388 in the beginning of the treatment (Fig. 3D, Table 1).

**Fig. 4.**
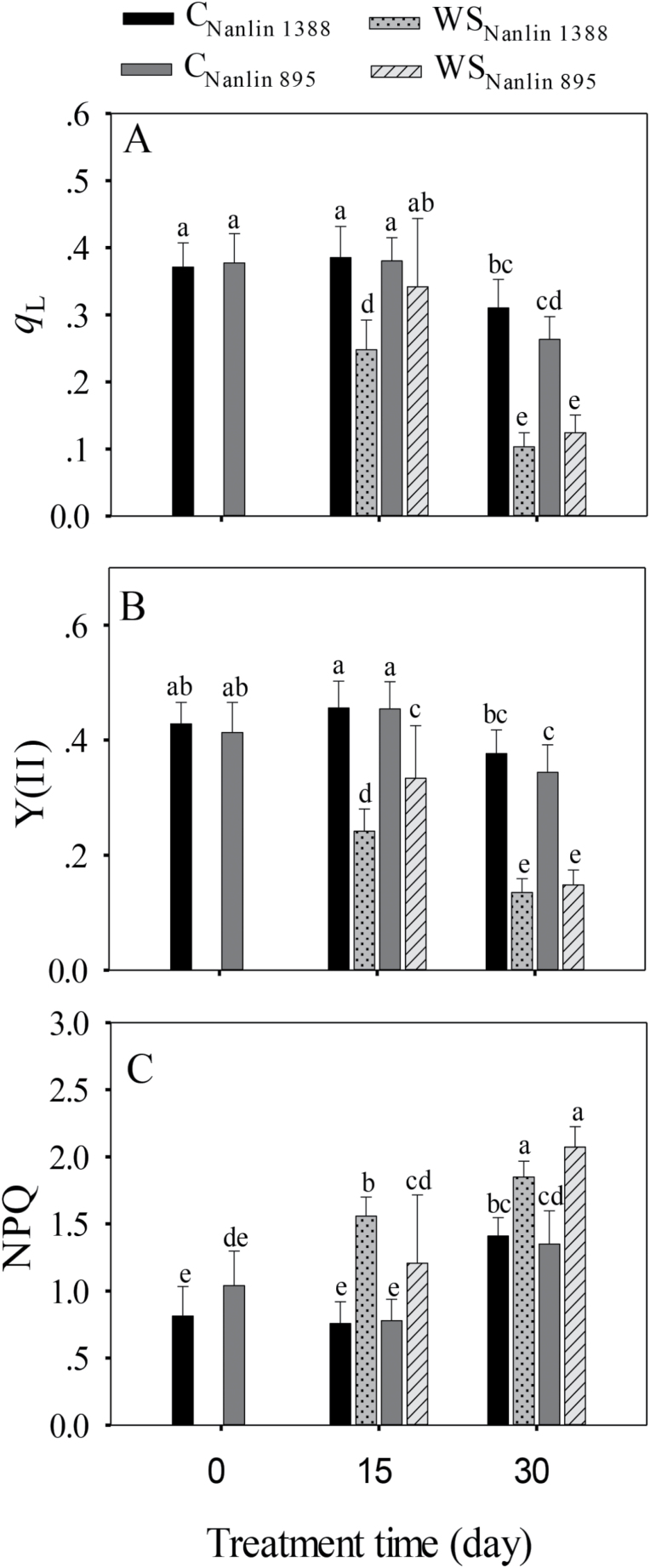
Effects of water stress on leaf chlorophyll fluorescence characteristics in two hybrid poplar clones, Nanlin 1388 (tolerant to water stress) and Nanlin 895 (tolerant to heat stress). C, control, WS, water stress (Fig. 1 for the experimental design and environmental conditions during the experiment). (A) Photochemical quenching, *q*_L_, (B) effective quantum yield of PSII, Y(II), and (C) non-photochemical quenching, NPQ. During the measurements, leaf temperature was set to 30 °C, and ambient CO_2_ concentration was 400 μmol mol^−1^. Data are means (± SD), *n* = 12–18. Different letters indicate significant differences between means as determined by one-way ANOVA followed by Duncan’s multiple range test (*P*<0.05).

The chlorophyll fluorescence variables *q*_L_ (eqn 2) and Y(II) (eqn 4), which characterize the use of light energy in photosynthesis, were affected by the drought treatment and treatment time similarly to the gas exchange characteristics (Fig. 3A–C versus Fig. 4A, B). Both *q*_L_ and Y(II) decreased with treatment time, and the magnitude of the decrease was greater in drought-stressed plants than in the controls (Table 1, Fig. 4A, B); the values in control plants at 15 d were the same as those at 0 d, but *q*_L_ and Y(II) declined significantly from 15 d to 30 d in both drought-stressed and control plants. Drought initially had a greater effect on the Nanlin 1388 plants, but the differences between the clones had vanished at 30 d (significant three-way interactions in Table 1). Changes in NPQ were generally opposite to those observed in *q*_L_ and Y(II) (Fig. 4C, Table 1).

### Changes in antioxidant enzyme activities under drought stress and long-term hot weather

**Fig. 5.**
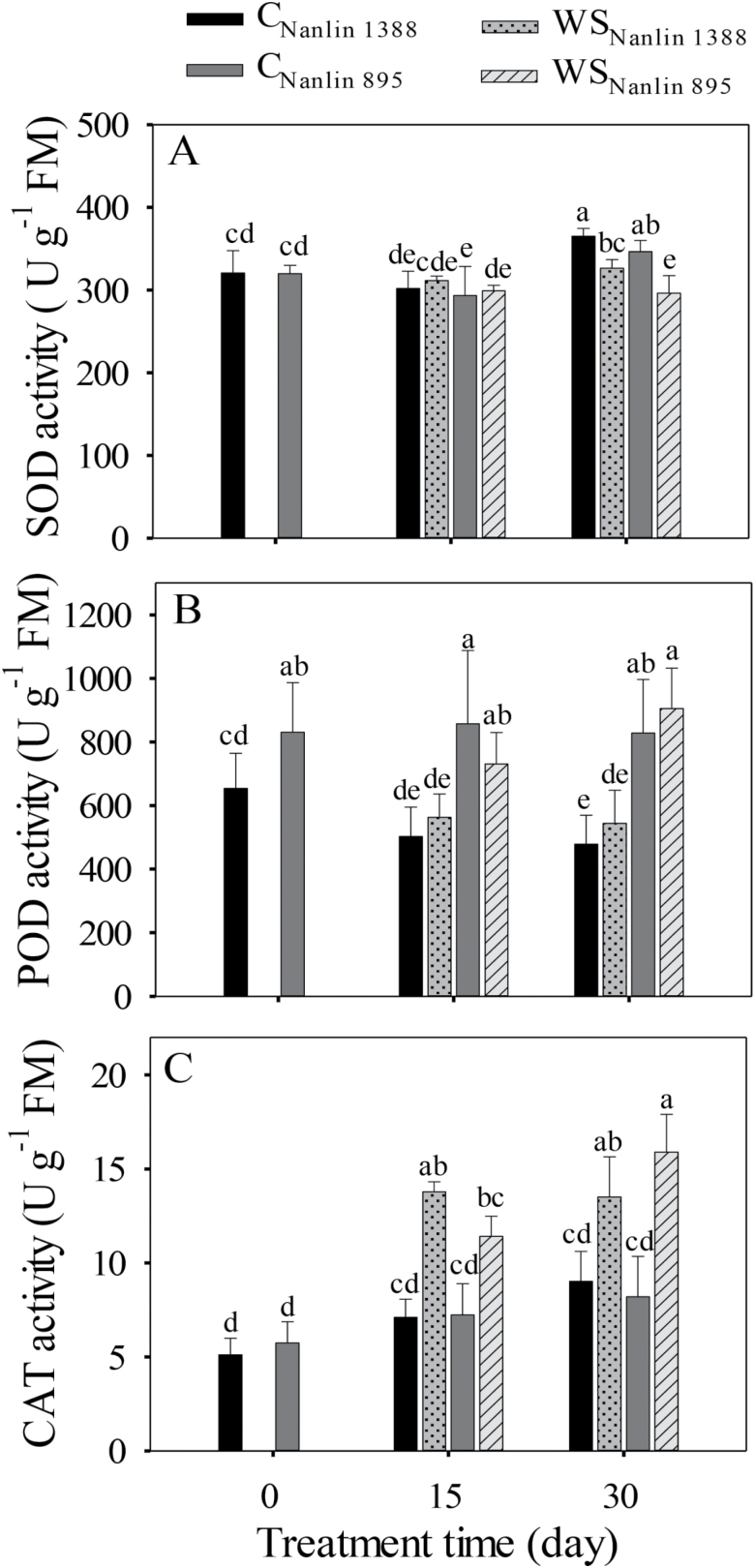
Effects of water stress on the activities of antioxidant enzymes in the leaves of two hybrid poplar clones, Nanlin 1388 (tolerant to water stress) and Nanlin 895 (tolerant to heat stress). C, control, WS, water stress (Fig. 1 for the experimental design and environmental conditions during the experiment). (A) Superoxide dismutase, SOD, (B) peroxidase, POD, and (C) catalase, CAT. Data are means (± SD), *n* = 12–18. Different letters indicate significant differences between means as determined by one-way ANOVA followed by Duncan’s multiple range test (*P*<0.05).

Drought and treatment time had relatively minor effects on superoxide dismutase (SOD) activity, with no significant main effect of drought observed at 15 d in either clone, although drought significantly decreased SOD activity at 30 d compared to the controls, particularly in Nanlin 895 (Fig. 5A, Table 1). No clear effects of drought and time of treatment were observed on peroxidase (POD) activity, but it was higher in Nanlin 895 than in Nanlin 1388 (Fig. 4B, Table 1). In both clones, catalase (CAT) activity was increased by drought stress at both 15 d and 30 d (Fig. 5C, Table 1). In addition, CAT activity was moderately increased in well-watered plants at later stages of treatment compared with the beginning of the treatment (Table 1).

### Relationships among isoprene emission, and photosynthetic characteristics, and antioxidant enzyme activities

**Fig 6.**
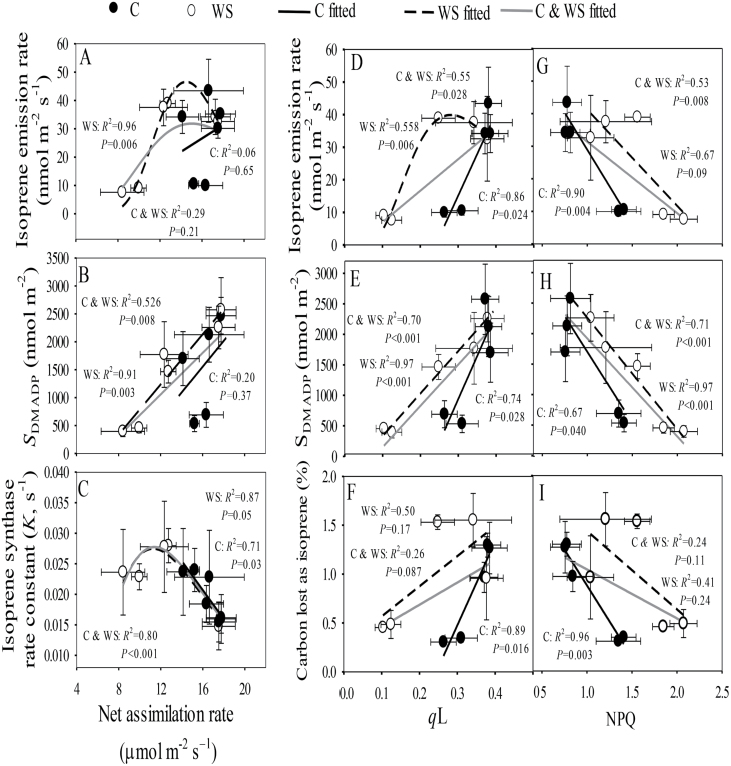
Relationships among isoprene emission and photosynthetic characteristics in the leaves of two hybrid poplar clones in response to water stress over a 30-d period. C, control, WS, water stress (Fig. 1 for the experimental design and environmental conditions during the experiment). Data for the two clones, Nanlin 1388 (tolerant to water stress) and Nanlin 895 (tolerant to heat stress), are pooled as the clone effect on the correlations was minor (Table 1). (A–C) Relationships between net assimilation rate and (A) isoprene emission rate, (B) size of pool of DMADP, *S*_DMADP_, and (C) the isoprene synthase reaction rate constant, *K*. (D–F) Relationships between photochemical quenching, *q*_L_, and (D) isoprene emission rate, (E) *S*_DMADP_, and (F) percentage of carbon lost as isoprene. (G–I) Relationships between non-photochemical quenching, NPQ, and (G) isoprene emission rate, (H) *S*_DMADP_, and (I) percentage of carbon lost as isoprene. Data are means (± SD), *n* = 4–6 for gas exchange and *n* = 12–18 for chlorophyll fluorescence. Regression coefficients are shown together with the *P*-values.

Analysis of covariation of isoprene emission and photosynthetic characteristics indicated that *I* was curvilinearly correlated with *A*_n_ only in drought-stressed plants (Fig. 6A), reflecting the fact that there was a greater decline in *I* than in *A*_n_ in well-watered plants during the course of the hot weather (Fig. 2A versus Fig. 3A). *S*_DMADP_ also scaled positively with *A*_n_ for drought-stressed plants and for all the data pooled, with the correlations being close to linear (Fig. 6B). IspS activity initially increased with increasing *A*_n_ in water-stressed plants, and then decreased both in water-stressed and control plants (Fig. 6C). As the variation in *C*_i_ broadly reflected the variation in *A*_n_ (Fig. 3A, B), the relationships between isoprene emission and *C*_i_ were similar to those with *A*_n_ (data not shown). The correlations between isoprene emission characteristics and the fraction of open PSII centers, *q*_L_ (Fig. 6D–F), and the effective quantum yield of PSII, Y(II) (data not shown), were similar to the correlations with *A*_n_. These correlations were strongest for control plants, but no uniform relationships between isoprene emission characteristics and *q*_L_ (Fig. 6D-F) and Y(II) were observed across the treatments. *I* and *S*_DMADP_ were negatively correlated with NPQ (Fig. 6G, H).

**Fig. 7.**
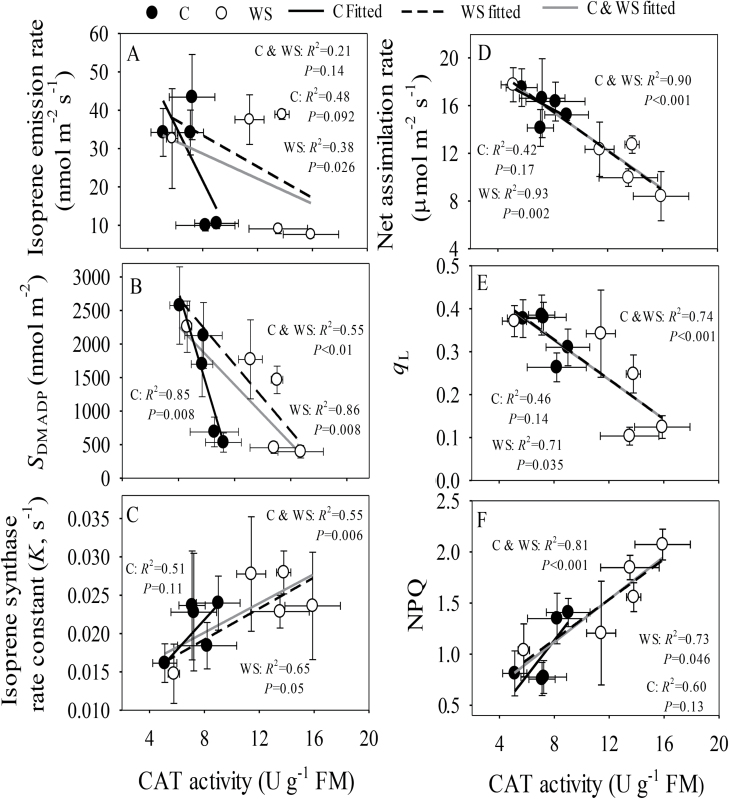
Relationships between catalase activity and isoprene emission and photosynthetic characteristics in the leaves of two hybrid poplar clones in response to water stress over a 30-d period. C, control, WS, water stress (Fig. 1 for the experimental design and environmental conditions during the experiment). Data for the two clones, Nanlin 1388 (tolerant to water stress) and Nanlin 895 (tolerant to heat stress), are pooled as the clone effect on the correlations was minor (Table 1). (A–C) Relationships between catalase (CAT) activity and (A) isoprene emission rate, (B) size of pool of DMADP, *S*_DMADP_, and (C) the isoprene synthase reaction rate constant. (D–F) Relationships between CAT activity and (D) net assimilation rate, (E) photochemical quenching, *q*_L_, and (F) non-photochemical quenching, NPQ. Data are means (±SD), *n* = 4–6 for gas exchange and *n* = 12–18 for chlorophyll fluorescence. Regression coefficients are shown together with the *P*-values.

*I* and *S*_DMADP_ were negatively correlated with CAT activity whilst *K* was positively correlated (Fig. 7A–C), and the slopes of these relationships differed between the water-stressed and control treatments (Fig. 7A—C). In contrast, uniform linear relationships across the treatments were observed between the photosynthetic characteristics and CAT activity (Fig. 7D–F).

## Discussion

### Two ecologically diverse clones performed similarly under drought stress and hot weather

Although the capacity for isoprene emission is considered as a trait improving tolerance to abiotic stresses, the evidence for a possible contribution of isoprene emission to the different thermotolerance and drought resistance characteristics of the two poplar clones examined in our study was very limited. Indeed, the responses of isoprene emission and photosynthetic characteristics to water stress combined with hot weather were similar between the two clones (Table 1, Figs 2, 3). Some differences between the two clones in isoprene emission characteristics were found, mainly after 15 d of treatment (Fig. 2). In contrast, after 30 d of treatment there were no differences in isoprene emission traits among the clones, except for isoprene synthase rate constant, *K*, that characterizes the biochemical capacity for isoprene emission (Fig. 2). At that time-point, *I*, *S*_DMADP_, and carbon lost as isoprene had all decreased to very low levels, indicating that the effects of prolonged exposure to high ambient temperature alone and in combination with long-term, severe water stress were different from the responses to moderate or short-time stress. Thus, in our study the clones with different ecological potentials only moderately differed in their isoprene emission responses to the severe, combined stresses. Previous work has demonstrated that the abiotic stress resistance of the two clones differs in several other ways in addition to their different tolerances to drought stress and heat stress. Compared with Nanlin 895, it has been reported that Nanlin 1388 has greater tolerance to cadmium and salt stress due to superior osmotic, ion homeostasis, and detoxification mechanisms (Wang *et al.*, 2016; Bi *et al.*, 2020). Hence, Nanlin 1388 might perform better under drought as a result of its enhanced capacity for osmotic adjustment. The presence of alternative stress-tolerance mechanisms might therefore explain why the clonal differences in ecological potential were not reflected in differences in isoprene emissions under stress.

Similar to our observations on isoprene emission and photosynthetic characteristics, the activities of antioxidant enzymes differed little between the clones at the different measurement time-points and under the different stresses (Figs 3–5). However, compared with Nanlin 895, water-stressed Nanlin 1388 had lower *q*_L_ and Y(II) after 15 d of treatment (Fig. 4A, B) and lower POD activity at both measurement time-points (Fig. 5B). This further highlights the lack of major differences between the two clones in their responses to severe combined stress, and our results are overall consistent with previous evidence that poplars, which are typical early successional species in boreal and temperate ecosystems, have only moderate drought and heat resistance (Laanisto and Niinemets, 2015).

### Implications of changes in isoprene emissions for direct protection of leaf photosynthetic activity

Our data collectively suggested that the regulation of isoprene emissions under severe combined stresses in the two clones was independent of their ecological potentials. We observed that after long-term hot weather (~1 month; Fig. 1), isoprene emissions were reduced to a very low level in both water-stressed and non-water-stressed plants (~75% decrease; Fig. 2A). Whilst several previous studies have shown that a severe water stress reduces emissions (Pegoraro *et al.*, 2004; Fortunati *et al.*, 2008; Brilli *et al.*, 2013), the major reduction we observed in the well-watered plants is surprising. As noted in the Introduction, isoprene emission can acclimate relatively rapidly to increases in air temperature. However, we found that sustained hot weather either alone or combined with severe drought reduced isoprene emission and the size of the pool of its substrate DMADP (Fig. 2A, B), although isoprene synthase activity was increased in both the water-stressed and non-water-stressed plants at both 15 d and 30 d after start of water stress treatment (Fig. 2C). Indeed, at 30 d after the start of the treatment, the reduction in *S*_DMADP_ was much greater than the increase in IspS activity, leading to a major decrease in isoprene emission rate across all treatments, and suggesting that limited substrate supply was the main factor controlling isoprene emission under severe long-term abiotic stress. As the study was conducted under natural conditions, there were no plants without exposure to high temperature during the long-term drought. Nevertheless, as drought and heat often interact under natural conditions (Valladares and Pearcy, 1997; Niinemets, 2010), our experiment provides a snapshot that is characteristic of plant performance in stressful environments in the field. Further experiments under controlled conditions would be needed to separate the individual effects of heat and drought.

The functional significance of isoprene emission is still a matter of debate (Fini *et al.*, 2017; Sharkey and Monson, 2017). Since isoprene emission improves plant resistance to abiotic stress, the key question is why isoprene emission decreased to a very low level under long-term severe stress when it would be most needed to protect the photosynthetic apparatus? The presence of contrasting short-term and long-term effects of hot weather on isoprene emissions would have important implications for understanding the role of isoprene as a potential antioxidant and an agent for improving leaf thermal tolerance. Isoprene concentrations in leaves with high isoprene emission rates and low *g*_s_ are only of the order of a few ppm (Singsaas *et al.*, 1997; Sun *et al.*, 2013b). Furthermore, the temperature optimum for isoprene emission is typically between 35–40 °C (Niinemets *et al.*, 1999; Rasulov *et al.*, 2010), while the optimum temperature for IspS is relatively high, 45–50 °C (Monson *et al.*, 1992; Niinemets *et al.*, 1999; Rasulov *et al.*, 2010). However, sudden increases in temperature can occur in the field, for example light-flecks can elevate leaf temperature by 10–15 °C above the air temperature (Singsaas *et al.*, 1999; Hüve *et al.*, 2019), and the activity of IspS could thus increase temporarily for a few minutes. This would support high isoprene emission rates at the expense of the existing DMADP pool, and possibly also at the expense of the existing pool of the upstream metabolite 2-C-methyl-D-erythritol 2,4-cyclodiphosphate (MEcDP). Thus, a temporal enhancement of isoprene release can in principle lead to a local elevation in isoprene concentrations in the thylakoid and chloroplast envelope membranes until a steady-state is reached (Singsaas *et al.*, 1999). Isoprene could thus improve leaf thermal tolerance under fluctuating temperature conditions, as has been observed in several studies (Behnke *et al.*, 2007, 2013; Way *et al.*, 2011).

The situation is different under longer heat episodes exceeding several minutes, when the DMADP pool size decreases and leads to a reduction in isoprene emission. Isoprene build-up within the leaf interior is small under steady-state conditions, and it does not necessarily protect leaves from long-term heat stress. Recent studies have found that isoprene build-up under physiological conditions does not change the viscosity of thylakoid phospholipid membranes in spinach leaves (Harvey *et al.*, 2015; Harvey and Sharkey, 2016). As our results showed that isoprene emission progressively decreased under sustained hot temperatures (Fig. 2A), the possible protective role of isoprene under sustained hot weather is likely small.

**Fig. 8.**
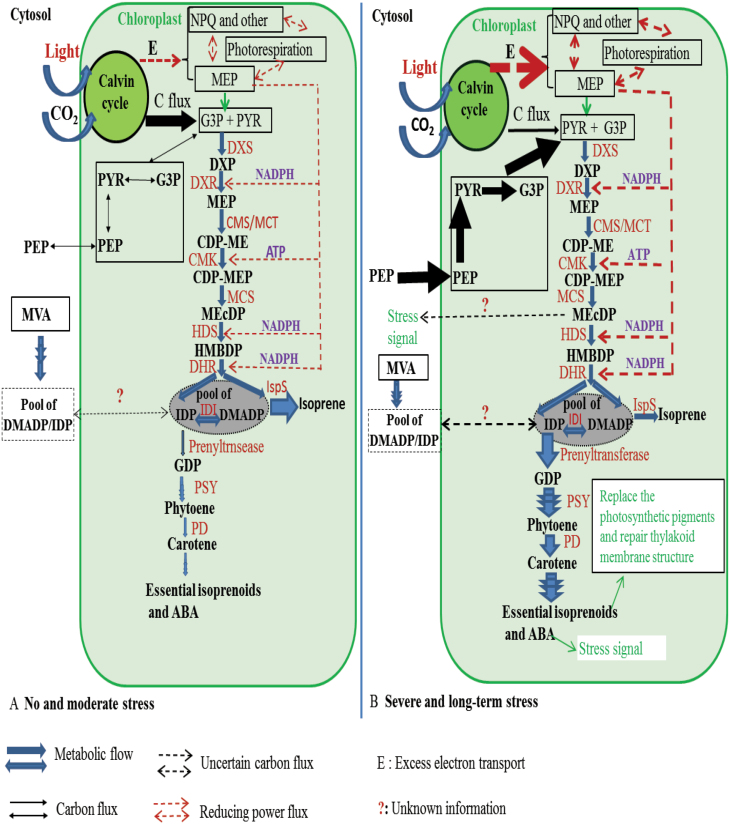
Schematic overview of the regulation of chloroplastic isoprene formation via the MEP pathway in (A) non-stressed and moderately stressed leaves and (B) leaves under severe stress. Arrows represent carbon or energy fluxes. Metabolites: PEP, phosphoenolpyruvic acid; MEP, 2-C-methyl-D-erythritol 4-phosphate; MVA, mevalonic acid; PYR, pyruvate; G3P, glyceraldehyde-3-phosphate; DXP, 1-deoxy-D-xylulose 5-phosphate; MEP-CDP, 4-(cytidine 5’-diphospho)-2-C-methyl-D-erythritol 2-phosphate; MEcDP, 2-C-methyl-D-erythritol 2,4-cyclodiphosphate; HMBDP, 1-hydroxy-2-methyl-2-(*E*)-butenyl 4-diphosphate; GDP, geranyl diphosphate; ABA, abscisic acid. Enzymes: DXS, DXP synthase; DXR, DXP reductoisomerase; CMS/MCT, MEP cytidylyltransferase; CMK, MEcDP kinase; MCS, MEcDP synthase; HDS, HMBDP synthase; HDR, HMBDP reductase; IDI, isopentenyl diphosphate isomerase; PSY, phytoene synthase; PD, phytoene desaturase.

The biosynthesis of isoprene and essential isoprenoids, such as carotenoids, phytol residues of chlorophylls, and plastoquinone, share the common substrates DMADP and IDP (Lichtenthaler, 2000, 2007; Banerjee and Sharkey, 2014; Sharkey and Monson, 2017) ). Under increased stress, direct carbon input from photosynthesis becomes increasingly limited, but chloroplast isoprenoid synthesis might be more strongly reliant on chloroplast starch and cytosolic carbon sources, provided that the key enzymes responsible for isoprenoid synthesis remain active, as was observed for isoprene synthesis in our study (Fig. 2C). On the other hand, severe stress typically leads to damage of the photosynthetic apparatus due to over-excitation of reaction centers and excess light interception by pigment–protein complexes. Given the higher substrate affinity of the enzymes responsible for large isoprenoid synthesis compared with isoprene synthase, under stress, DMADP and IDP could be shunted for rapid use for biosynthesis of carotenoids, phytol residues of chlorophylls, zeaxanthin, and abscisic acid (ABA), as hypothesized in Fig. 8. This is consistent with the hypothesis that the presence of isoprene emissions in broadleaved deciduous shade-intolerant species with high rates of photosynthesis allows plastic reconfiguration of isoprenoid synthesis under stress to maintain the integrity of photosynthetic machinery.

### Scaling of isoprene emissions with the supply of photosynthetic metabolites and energy

Drought led to classic changes in leaf photosynthetic characteristics including reductions in stomatal conductance (*g*_s_), intercellular CO_2_ concentration (*C*_i_), net assimilation rate (*A*_n_) (Fig. 3), decreases in the openness of PSII centers (*q*_L_, characterizing the oxidation state of the primary electron acceptor *Q*_A_), reductions in effective PSII quantum yield [Y(II)], and increases in non-photochemical quenching (NPQ) (Fig. 4), and these changes became greater with the treatment time. However, hot weather alone did not affect the chlorophyll fluorescence characteristics at day 15, indicating that at this stage, photorespiratory use of photosynthetic energy replaced the reduced use of NADPH and ATP in carbon assimilation in leaves with reduced *C*_i_. In addition, in the well-watered plants, the decrease in photosynthetic traits at day 30 was much less than in the combined hot weather and drought treatment (Figs 3, 4). The modifications in photosynthetic characteristics on both measurement dates were different from the changes in isoprene emission, which responded much more strongly to hot weather alone (Fig. 2A versus Figs. 3, 4). Furthermore, isoprene emission was more strongly reduced in the combined hot weather/drought stress treatment at day 30 than the leaf photosynthetic characteristics. Previous studies have found that moderate water stress stimulates isoprene emissions (Brüggemann and Schnitzler, 2002; Pegoraro *et al.*, 2004; Brilli *et al.*, 2007; Fortunati *et al.*, 2008; Brunetti *et al.*, 2015; Tattini *et al.*, 2015); however, the emissions decrease as drought becomes more severe, and this has been associated with limited availability of carbon and photosynthetic energy for isoprene emission (Brilli *et al.*, 2007; Tattini *et al.*, 2014, 2015). Our results for water-stressed leaves, especially at the end of the treatment period, could be interpreted on the basis of limited carbon availability.

In non-water-stressed plants, the size of the DMADP pool decreased upon exposure to hot weather without any concomitant modifications in most of the leaf photosynthetic characteristics (Fig. 2B versus Figs 3, 4), indicating uncoupling between isoprene emission and photosynthetic characteristics under prolonged high temperature stress. Our previous studies have demonstrated that the isoprene emission rate is strongly correlated with photosynthetic energy supply in non-stressed hybrid poplars measured under ambient and elevated CO_2_ concentration (Sun *et al.*, 2012; 2013*a*; Grote *et al.*, 2014; Morfopoulos *et al.*, 2014; Niinemets and Sun, 2015; Niinemets *et al.*, 2015). However, because of the different responses of isoprene emission and photosynthesis to short and long-term stress, there were no uniform relationships between the rates of photosynthesis and isoprene emission in the current study (Figs 6, 7). In particular, non-water-stressed plants at day 30 deviated the most from the general relationships (Figs 6, 7), indicating that the share of carbon and photosynthetic energy for isoprene emission was reduced for these plants at the end of the treatment. This result is difficult to interpret and might indicate overall suppression of the activity of the MEP pathway under heat stress. On the other hand, the low pool size of DMADP available for isoprene emission might also indicate increased activity of competing biochemical processes that were required to repair the damage to the photosynthetic apparatus when the plants experienced severe or long-term abiotic stress (Fig. 8; see next section).

### Is a reduction in isoprene emission rate consistent with isoprene playing a role in maintaining an active MEP pathway?

In addition to isoprene, DMADP and IDP are also the substrates for all the downstream products of the MEP pathway, including carotenoids, phytol residues of chlorophylls, ubiquinone, tocopherols, and ABA (Lichtenthaler, 2000, 2007; Vranová *et al.*, 2013; Banerjee and Sharkey, 2014). The components of the photosynthetic light-harvesting pigment–protein complexes can suffer damage under different abiotic stresses, and it is expected that pigment synthesis is enhanced in response to stress (Beisel *et al.*, 2010, 2011). Carotenoids of the xanthophyll cycle play an important role in the dissipation of excess excitation energy, and both xanthophylls and tocopherols can serve as antioxidants; hence, their synthesis is expected to be enhanced under stress (Bilger and Björkman, 1990; Havaux *et al.*, 2000, 2007; Müller *et al.*, 2001; Haupt-Herting and Fock, 2002; Niinemets *et al.*, 2003; Lichtenthaler, 2007; Vranová *et al.*, 2013). It has been proposed that the primary role of isoprene emission is to maintain the activity of the MEP pathway, thus allowing the rapid use of DMADP when it is needed for the synthesis of essential isoprenoids (Rosenstiel *et al.*, 2003; Owen and Peñuelas, 2005; de Souza *et al.*, 2018). Because the Michaelis–Menten constant, *K*_m_, for DMADP is large (Schnitzler *et al.*, 1996; Rasulov *et al.*, 2014), isoprene-emitting species maintain a very big DMADP pool (Nogués *et al.*, 2006). A greater affinity of larger isoprenoid synthesis for DMADP implies that even if the size of the DMADP pool and rate of isoprene synthesis are reduced, synthesis of essential isoprenoids can still occur with a high rate. Our observation of an apparent reduction in *S*_DMADP_ under hot weather in both water-stressed and non-water-stressed plants is consistent with the hypothesis of a greater capacity to support essential isoprenoid synthesis under stress in isoprene-emitting plants (Fig. 8). To confirm this, further studies are needed to assess the changes in the rates of synthesis of non-volatile isoprenoids during stress development.

### Changes in antioxidative enzyme activity and their relationships with isoprene emission and photosynthesis

In plants, most reactive oxygen species, such as the superoxide radical (O_2_^−^), hydrogen peroxide (H_2_O_2_), and singlet oxygen (^1^O_2_), are produced by photosynthesis in chloroplasts as a result of environmental stress (Müller *et al.*, 2001; Havaux *et al.*, 2007; Slesak *et al.*, 2007; Johnson, 2011). Located in the chloroplast, SOD is a key antioxidant enzyme in the Mehler reaction that quenches O_2_^−^ (Makino *et al.*, 2002; Maruta *et al.*, 2016). SOD rapidly converts this highly active oxidant to the less potent H_2_O_2_, which in turn is converted to H_2_O and O_2_ by the chloroplastic POD enzyme. Because of its high efficiency, chloroplastic SOD is rarely considered to be a limiting factor in this process (Bowler *et al.*, 1994; Bian and Jiang, 2009). However, POD has been considered as a bottleneck in the Mehler reaction (Makino *et al.*, 2002; Meloni *et al.*, 2003; Slesak *et al.*, 2007). CAT is a key enzyme responsible for scavenging of excess H_2_O_2_ produced in peroxisomes, and its activity has been associated with the rate of photorespiration in C_3_ plants under stress (Makino *et al.*, 2002; Slesak *et al.*, 2007; Joliot and Johnson, 2011; Karuppanapandian *et al.*, 2011). We found that SOD activity was not affected at 15 d after the start of the treatment, and POD activity was either not altered (clone Nanlin 895) or was even reduced (Nanlin 1833) in both stressed and non-stressed plants at 15 d and 30 d (Fig. 5A, B). This suggested that the Mehler reaction was probably responsible for the use of only a small part of the excess excitation energy from the photosynthetic electron transport chain, although the functions of Mehler reaction remain controversial (Ogren, 1984; Makino *et al.*, 2002; Joliot and Johnson, 2011; Maruta *et al.*, 2016).

In contrast to SOD and POD, CAT activity increased in response to hot weather and water stress (Table 1), especially in water-stressed plants (Fig. 5C), indicating that CAT-dependent antioxidative processes, including photorespiration, were up-regulated. Increases in CAT activity were strongly correlated with reductions in *A*_n_ and PSII photochemistry and with increases in NPQ (Fig. 7D–F), indicating that CAT activity responded sensitively to stress-dependent changes in leaf photosynthetic activity. The increases in CAT activity are in agreement with enhanced photorespiration and consumption of excess electrons from the photosynthetic electron transport chain, especially under stress when CO_2_ supply is limited. This allows H_2_O_2_ to be maintained at a low level and contributes to cellular redox homeostasis (Ogren, 1984; Wingler *et al.*, 2000; Meloni *et al.*, 2003; Ruban, 2016).

CAT activity was also correlated with the isoprene emission characteristics, but the correlations varied among the different treatments (Fig. 7A–C). Although any stress would be expected to enhance the overall leaf antioxidative capacity, CAT is active in the liquid phase, while isoprene can serve as an antioxidant in leaf lipid and gas phases. However, as already noted, even in leaves with closed stomata, the build-up of isoprene is relatively low, and therefore its capacity to scavenge reactive oxygen species through direct reactions is also limited. Given the inherent limitation of isoprene in protecting against long-term heat events as discussed above, it is plausible that a sustained high-temperature environment leads to enhanced leaf lipid-phase antioxidative capacity via enhanced production of tocopherols and xanthophylls (Abbasi *et al.*, 2007; Zhang *et al.*, 2011; Fritsche *et al.*, 2017). Because of the high cost of sustained isoprene release, these non-volatile antioxidants could contribute more efficiently to the maintenance of the integrity of the photosynthetic machinery (Brunetti *et al.*, 2015; Harvey *et al.*, 2015; Harris *et al.*, 2016; Harvey and Sharkey, 2016). Further studies are needed to assess the changes in lipid-phase antioxidative capacity upon exposure of plants to sustained high temperatures.

### Conclusions

Our results indicated that isoprene synthase activity in two poplar clones acclimated to hot weather in both water-stressed and non-water-stressed plants, whereas isoprene emission did not. This reflected depletion of precursor pools for isoprene synthesis. In addition, isoprene emission was more sensitive than carbon assimilation and PSII photochemistry to severe prolonged stress. As the result, isoprene emission was partly uncoupled from leaf photosynthetic characteristics. Thus, our results suggested that isoprene played a minor role in protection against sustained heat stress in poplar. However, the turnover of larger, non-volatile isoprenoids, specifically isoprenoid antioxidants and pigment components of the photosynthetic machinery, can increase under stress when the photosynthetic apparatus becomes damaged under adverse conditions. The results of our study support the hypothesis that isoprene emission as a trait could contribute to maintaining the activity of the MEP pathway for the synthesis of non-volatile isoprenoid antioxidants and photosynthetic pigments.

Our results also have major implications for modelling vegetation isoprene emissions. Acclimation of isoprene emissions to temperature environment has been incorporated in global models of isoprene emissions (Guenther *et al.*, 2006, 2012), but these models assume that high temperatures, independent of the duration of the heat event, have a positive effect on foliage isoprene emission. However, our results suggest that sustained hot weather periods might actually strongly suppress isoprene emissions. As such sustained periods of heat are expected to become more severe under future climate scenarios, more experimental research is needed to gain insights into the regulation of isoprene emissions under these conditions.

## Supplementary data

The following supplementary data are available at *JXB* online.

Table S1. Daily maximum and minimum temperatures at the experimental site during the experiment.

eraa415_suppl_Supplementary-Table-S1

## Data Availability

The data supporting the findings of this study are available from the corresponding author, Zhihong Sun, upon request.
