# Supplementary material for: Responses of isoprene emission and photochemical efficiency to severe drought combined with prolonged hot weather in hybrid Populus
Source: J Exp Bot. 2020 Sep 30;71(22):7364–81. doi: 10.1093/jxb/eraa415 (PMC7906789; doi:10.1093/jxb/eraa415)
Supplement: eraa415_suppl_Supplementary-Table-S1 [file eraa415_suppl_supplementary-table-s1.pdf]

Table S1. Daily temperature (℃) from March 15 to August 14, 2017 during the whole experiment. Data was obtained from the weather station of Lin'an, Hangzhou city (http://lishi.tianqi.com/linan/index.html).

Date	Daily temperature		Date	Daily temperature		Date	Daily temperature		Date	Daily temperature		Date	Daily temperature	
	Max	Min		Max	Min		Max	Min.		Max	Min		Max	Min.
15-Mar-17	14	5	15-Apr-17	31	16	15-May-17	24	17	15-Jun-17	27	17	15-Jul-17	36	25
16-Mar-17	12	4	16-Apr-17	29	18	16-May-17	22	13	16-Jun-17	26	17	16-Jul-17	37	25
17-Mar-17	15	8	17-Apr-17	24	15	17-May-17	26	13	17-Jun-17	28	17	17-Jul-17	37	26
18-Mar-17	13	8	18-Apr-17	30	14	18-May-17	30	15	18-Jun-17	28	20	18-Jul-17	36	25
19-Mar-17	11	9	19-Apr-17	29	18	19-May-17	30	17	19-Jun-17	25	21	19-Jul-17	37	25
20-Mar-17	13	8	20-Apr-17	25	15	20-May-17	29	17	20-Jun-17	27	21	20-Jul-17	37	26
21-Mar-17	14	7	21-Apr-17	23	11	21-May-17	30	17	21-Jun-17	26	21	21-Jul-17	38	26
22-Mar-17	9	6	22-Apr-17	22	9	22-May-17	30	19	22-Jun-17	28	23	22-Jul-17	39	26
23-Mar-17	13	8	23-Apr-17	25	10	23-May-17	26	18	23-Jun-17	28	22	23-Jul-17	39	26
24-Mar-17	10	7	24-Apr-17	27	14	24-May-17	23	14	24-Jun-17	24	21	24-Jul-17	40	26
25-Mar-17	17	6	25-Apr-17	25	15	25-May-17	28	14	25-Jun-17	26	20	25-Jul-17	40	26
26-Mar-17	19	4	26-Apr-17	18	11	26-May-17	29	16	26-Jun-17	27	21	26-Jul-17	39	26
27-Mar-17	21	7	27-Apr-17	21	9	27-May-17	31	17	27-Jun-17	29	21	27-Jul-17	39	26
28-Mar-17	20	9	28-Apr-17	26	11	28-May-17	32	17	28-Jun-17	24	20	28-Jul-17	38	26
29-Mar-17	20	10	29-Apr-17	28	12	29-May-17	32	18	29-Jun-17	29	22	29-Jul-17	37	26
30-Mar-17	13	8	30-Apr-17	29	14	30-May-17	32	21	30-Jun-17	29	23	30-Jul-17	33	26
31-Mar-17	13	5	1-May-17	29	16	31-May-17	31	22	1-Jul-17	33	24	31-Jul-17	35	26
1-Apr-17	5	0	2-May-17	22	15	1-Jun-17	28	20	2-Jul-17	32	24	1-Aug-17	30	25
2-Apr-17	22	7	3-May-17	24	17	2-Jun-17	30	20	3-Jul-17	31	24	2-Aug-17	32	26
3-Apr-17	23	11	4-May-17	23	14	3-Jun-17	30	19	4-Jul-17	32	24	3-Aug-17	35	26

4-Apr-17	25	13	5-May-17	27	16	4-Jun-17	28	19	5-Jul-17	34	24	4-Aug-17	37	26
5-Apr-17	26	16	6-May-17	24	15	5-Jun-17	21	19	6-Jul-17	35	25	5-Aug-17	36	26
6-Apr-17	26	17	7-May-17	26	16	6-Jun-17	25	18	7-Jul-17	35	26	6-Aug-17	35	26
7-Apr-17	21	14	8-May-17	21	16	7-Jun-17	29	19	8-Jul-17	34	25	7-Aug-17	37	27
8-Apr-17	26	14	9-May-17	27	14	8-Jun-17	32	21	9-Jul-17	33	25	8-Aug-17	36	26
9-Apr-17	14	9	10-May-17	32	16	9-Jun-17	29	22	10-Jul-17	32	24	9-Aug-17	30	25
10-Apr-17	13	9	11-May-17	33	18	10-Jun-17	30	21	11-Jul-17	32	24	10-Aug-17	34	25
11-Apr-17	15	9	12-May-17	23	15	11-Jun-17	26	20	12-Jul-17	35	25	11-Aug-17	35	25
12-Apr-17	19	7	13-May-17	32	16	12-Jun-17	21	18	13-Jul-17	36	25	12-Aug-17	30	25
13-Apr-17	24	9	14-May-17	31	17	13-Jun-17	20	19	14-Jul-17	35	25	13-Aug-17	35	26
14-Apr-17	28	13				14-Jun-17	25	17				14-Aug-17	33	25
